# Physical activity and the mediating effect of fear, depression, anxiety, and catastrophizing on pain related disability in people with chronic low back pain

**DOI:** 10.1371/journal.pone.0180788

**Published:** 2017-07-07

**Authors:** Paul W. M. Marshall, Siobhan Schabrun, Michael F. Knox

**Affiliations:** School of Science and Health, Western Sydney University, Penrith South, New South Wales, AUSTRALIA; Southeast University Zhongda Hospital, CHINA

## Abstract

**Background:**

Chronic low back pain is a worldwide burden that is not being abated with our current knowledge and treatment of the condition. The fear-avoidance model is used to explain the relationship between pain and disability in patients with chronic low back pain. However there are gaps in empirical support for pathways proposed within this model, and no evidence exists as to whether physical activity moderates these pathways.

**Methods:**

This was a cross-sectional study of 218 people with chronic low back pain. Multiple mediation analyses were conducted to determine the role of fear, catastrophizing, depression, and anxiety in the relationship between pain and disability. Separate analyses were performed with physical activity as the moderator. Individuals were classified as performing regular structured physical activity if they described on average once per week for > 30-minutes an activity classified at least moderate intensity (≥ 4–6 METs), activity prescribed by an allied health professional for their back pain, leisure time sport or recreation, or self-directed physical activity such as resistance exercise.

**Results:**

Fear, catastrophizing, and depression significantly mediated the relationship between pain and disability (p<0.001). However the mediating effect of catastrophizing was conditional upon weekly physical activity. That is, the indirect effect for catastrophizing mediating the relationship between pain and disability was only significant for individuals reporting weekly physical activity (*B* = 1.31, 95% CI 0.44 to 2.23), compared to individuals reporting no weekly physical activity (*B* = 0.21, 95% CI -0.50 to 0.97). Catastrophizing also mediated the relationship between pain and fear (*B* = 0.37, 95% CI 0.15 to 0.62), with higher scores explaining 53% of the total effect of pain on fear.

**Conclusions:**

These results support previous findings about the importance of fear and depression as factors that should be targeted in low back pain patients to reduce back pain related disability. We have also extended understanding for the mediating effect of catastrophizing on back pain related disability. Back pain patients engaged with regular physical activity may require counselling with regards to negative pain perceptions.

## Introduction

Low back pain is the musculoskeletal condition with the greatest worldwide burden of disease, defined in terms of disability adjusted life years or years lived with disability [1]. The economic cost to society is considerable, with direct annual costs of treatment in Australia estimated to be $4.8 billion [2], and total treatment costs approximately $9 billion [3]. Despite both pain and disability being associated with a range of psychosocial and physical factors [4–7], the direct pathways that link pain and disability remain unclear. Thus clinicians and researchers are faced with difficulty designing targeted interventions to alleviate the burden of chronic back pain. One theoretical model developed to explain how pain leads to disability is the fear-avoidance model [8,9]. However, despite its popularity for explaining disability, and integration into clinical trials to provide measures of treatment action, there are gaps in empirical support for pathways within the fear-avoidance model.

The fear-avoidance model was originally proposed to explain how exaggerated pain perception was the consequence of a heightened fear of pain and avoidance of social and physical activities [10]. In 2000 the model was updated to suggest that pain may lead to catastrophic thinking, with the subsequent increase in fear and physical disuse contributing to disability and psychological distress [9]. Recently, paths within the fear-avoidance model have been examined in a systematic review of mediation studies [11]. Mediation is a type of statistical analysis that examines proposed causal mechanisms thought to explain the relationship between two variables. This review reported that fear and psychological distress, but not catastrophizing, mediate the relationship between pain and disability. However a number of gaps in the literature were identified with regards to support for causal paths within the fear-avoidance model. First, studies examining the mediating effect of catastrophizing were not sufficiently powered based on recommended sample sizes for this type of analysis (total of 3 studies reviewed, n = 234; range n = 64 to 103), and only one of these studies sampled from a chronic back pain population [12]. The low power, particularly for chronic back pain patients, may explain the disparity between the review conclusions and outcomes from intervention studies that suggest catastrophizing mediates the effect of various physical activity and treatment interventions on back pain related disability [13]. Second, no study examined the first proposed pathway of the fear-avoidance model, which suggests that catastrophizing mediates the relationship between pain and fear [9]. Finally, no information was provided about factors (e.g. physical activity, pain duration) that may moderate pathways within the fear-avoidance model.

In contrast to mediation, which quantifies the effect a potential explanatory variable (e.g. fear) has on the relationship between an exposure (e.g. pain) and outcome (e.g. disability), moderation is an analysis technique that examines whether an external condition influences such a relationship. Within the context of the fear-avoidance model and low back pain, the regular performance of a structured physical activity program (e.g. therapist guided exercise program, cardiorespiratory exercise, self-prescribed trunk exercises) is a potential moderating variable that has not been examined. Adherence to prescribed physical activity or exercise in low back pain patients is typically poor, with reports of non-adherence in 50 to 70% of patients [14,15]. Moreover, physical activity levels have a negative association with disability in patients with chronic low back pain [16]. While it is plausible to suggest that back pain patients who do not engage in regular physical activity exhibit greater fear-avoidance, thus explaining the relationship between higher levels of pain and disability, this has not been well examined.

Therefore we conducted this study to provide further empirical investigation of proposed pathways within the fear-avoidance model in patients with chronic low back pain. The specific objectives of this study were 1) to investigate whether catastrophizing, in combination with fear and psychological distress, mediated the relationship between pain and disability, 2) to investigate whether catastrophizing mediated the relationship between pain and fear, and 3) to examine whether engagement with regular structured physical activity moderated the indirect effect of catastrophizing, fear, and psychological distress on the relationship between pain and disability.

## Materials and methods

### Study design

This cross-sectional study with mediation analysis used data from people with chronic low back pain, and does not report any outcomes following specific treatment.

### Participants

This study was based on data collected from 218 consecutive participants (out of 394 people screened for inclusion) with chronic low back pain from the local community who attended the local University School of Science and Health research facility between June 2011 and July 2016 (Table 1; S1 Table). Sample size estimates for mediation analysis to achieve 0.8 power [17] were based on previous data for the mediating effect of fear and depression on the relationship between pain and disability (a and b pathways *B* = 0.40), and required a minimum of 71 participants. This study was not sufficiently powered to detect significant indirect effects when the a and b paths (exposure to mediator, mediator to outcome respectively) were small (*B* = 0.14). For example a small ‘a path’ but large ‘b path’ (*B* = 0.60) is suggested to require n = 365. All data collection procedures received ethical approval from the Western Sydney University Human Research Ethics Committee. Written informed consent was received from all participants prior to proceeding with data collection. Participants were eligible for the study if they were between the ages of 18 and 65 years, had pain and/or impairment attributed to the low back > 3-months, with symptoms reported between T12 to the gluteal folds that was not from a specific origin (as confirmed from previous back surgical history, spondylolisthesis, spinal stenosis, persistent referred pain symptoms into the lower leg). Other exclusion criteria included any surgery in the last 3 months, pregnancy in the last 12-months, diagnosed psychiatric or somatoform disorder, or any other neuromuscular or metabolic disease.

**Table 1:** Baseline characteristics of study participants (n = 218) and the sub-groups of people identified as reporting weekly physical activity (PA) or no PA. Unless otherwise stated all data are mean ± SD. The types of physical activity and number of participants reporting weekly performance of the activity type are provided. Some participants reported multiple types of activity, thus there is some overlap.

	n = 218	PA, n = 68	No PA, n = 150
Age (years)	36.2 ± 6.6	35.6 ± 7.0	36.5 ± 6.4
Female (%)	59.6	73.5	34.7
Duration of symptoms (years)	10.9 ± 7.4	10.2 ± 7.5	11.2 ± 7.4
Height (m)	1.71 ± 0.08	1.69 ± 0.07	1.71 ± 0.09
Weight (kg)	82.0 ± 14.5	78.3 ± 13.0	83.6 ± 15.0
Paid work (%)	68.3	54.4	74.7
Medication for back pain, last month (%)	29.8	19.1	34.7
Regular physical activity (%)	31.1		
• Cardiorespiratory, n		30	-
• Trunk strengthening/stabilization exercise, n		35	-
• Flexibility exercise, n		8	-
• Leisure time sport & recreation, n		6	-
• Resistance exercise, n		1	-
Oswestry disability index (ODI, 0–100%)	24.9 ± 13.6	17.6 ± 10.7	28.2 ± 13.5
Pain intensity—current (VAS-c, 0–10 cm)	3.6 ± 2.3	2.6 ± 2.1	4.1 ± 2.3
Pain intensity—worst last week (VAS-w, 0–10 cm)	5.5 ± 2.6	4.3 ± 2.6	6.0 ± 2.4
Anxiety (HADS-a, 0–21)	6.0 ± 3.4	5.4 ± 3.4	6.3 ± 3.4
Depression (HADS-d, 0–21)	4.1 ± 3.7	3.2 ± 2.8	4.5 ± 4.0
Catastrophizing (PCS, 0–52)	15.6 ± 12.6	9.9 ± 10.6	18.2 ± 12.6
Fear-avoidance—physical (FABQ, 0–24)	13.8 ± 5.6	11.8 ± 5.7	14.8 ± 5.3
Fear-avoidance—work (FABQ, 0–42)	11.3 ± 9.8	10.6 ± 9.7	11.5 ± 5.3

### Assessment

All information for this study was collected from participants at an in-person meeting that included: duration of pain and disability symptoms (months or years), age, height, weight, and employment status over the last 3-months. Further information collected related to activities pursued in the last month for management of their back pain including medication use, consultation with an allied health professional (e.g. physiotherapist, chiropractor, clinical exercise physiologist) or other types of treatment (e.g. remedial massage, acupuncture). We also collected information about the frequency, intensity, type, and time of physical activities performed in the last month.

Individuals were classified as performing regular structured physical activity if they described on average once per week for > 30-minutes (in one bout or accumulated over a day) an activity classified at least moderate intensity (≥ 4–6 METs) defined by the American College of Sports Medicine (e.g. walking approximately 5.km.h^-1^ or other cardiorespiratory exercise, mowing lawns [18]), activity prescribed by an allied health professional for their back pain (e.g. trunk focussed exercise, stretching), leisure time sport or other recreational pursuits (e.g. golf without a cart), or self-directed physical activity such as resistance exercise.

Self-report questionnaires were subsequently administered at the in-person meeting comprising measures to examine pathways within the fear-avoidance model.

### Disability

Self-perceived disability was measured with the Oswestry Low Back Pain Disability Index (ODI) [19]. The ODI is a 10-item questionnaire regarding how a patient’s low back pain affects different aspects of their life such as walking, sitting, standing, and lifting. Each item has 6 corresponding answers that are scored in severity from 0 to 5. The scores from the 10-items are summed (maximum total of 50), and expressed as a percentage (0 to 100%). Studies have shown the ODI to have good construct validity [20], internal consistency and reliability [21].

### Self-rated low back pain

A 10-cm VAS with “no pain” on the left side and “worst pain” on the right side was used to measure the current pain intensity (VAS-c), and worst pain intensity in the last week (VAS-w) [22]. The VAS has been found to have good construct validity [23] and reliability [24].

### Fear-avoidance beliefs

The Fear Avoidance Beliefs Questionnaire (FABQ) was used to examine patient’s beliefs about the potential harm of work or general physical activity to their back pain [25]. The FABQ has 16 items, each scored from 0 to 6. Higher numbers indicate increased levels of fear-avoidance beliefs. Two subscales within the FABQ have been identified, a 7-item work subscale score (FABQ-w, score range 0–42), and a 4-item physical activity subscale score (FABQ-p, score range 0–24). The internal consistency and test-retest reliability of the FABQ are high [26].

### Pain catastrophizing

The Pain Catastrophizing Scale (PCS) was used [27]. The PCS is a 13-item questionnaire developed to identify catastrophic thoughts or feelings in relation to painful experiences. The total score ranges from 0 to 52 and high scores indicate that more catastrophic thoughts or feelings are experienced. The internal consistency and test-retest reliability of the PCS are high [28,29].

### Anxiety and depression

Anxiety and depression was measured using the 14-item Hospital Anxiety and Depression Scale (HADS). There are 7-items each for anxiety and depression, with items scored from 0 to 3; higher scores indicate greater anxiety (HADS-a) or depression (HADS-d). The total score for each sub-scale ranges from 0 to 21 [30]. The HADS has good internal consistency [31], reliability [31], and validity [32,33].

### Data analysis

Multiple mediation analysis was performed according to recommended procedures [34–37] to examine whether the relationship between pain and disability was explained by fear, catastrophizing, depression, and anxiety. Highly correlated variables that indicate multicollinearity (r > 0.90), or variables that were not correlated with either pain or disability, were excluded from the subsequent mediation analyses based on recommendations for multivariate analyses [38]. Multicollinearity between pain, disability, fear, catastrophizing, depression, and anxiety was assessed by performing Pearson correlations (Table 2).

**Table 2:** Correlations (r-value) between measure of disability (ODI) and pain (VAS-c, VAS-w) with depression (HADS-d), anxiety (HADS-a), fear (FABQ-a, FABQ-w), and catastrophizing (PCS).

	ODI	VAS-c	VAS-w	HADS-d	HADS-a	FABQ-a	FABQ-w	PCS
ODI	1.0	.514**	.457**	.406**	.267**	.458**	.213**	.570**
VAS-c		1.0	.680**	.260**	.137*	.292**	.243**	.580**
VAS-w			1.0	.192**	.123	.315**	.150*	.474**
HADS-d				1.0	.579**	.089	.357**	.538**
HADS-a					1.0	.009	.223**	.374**
FABQ-a						1.0	.128	.347**
FABQ-w							1.0	.242**
PCS								1.0

** is p<0.01, and

* is p<0.05.

**Fig 1:**
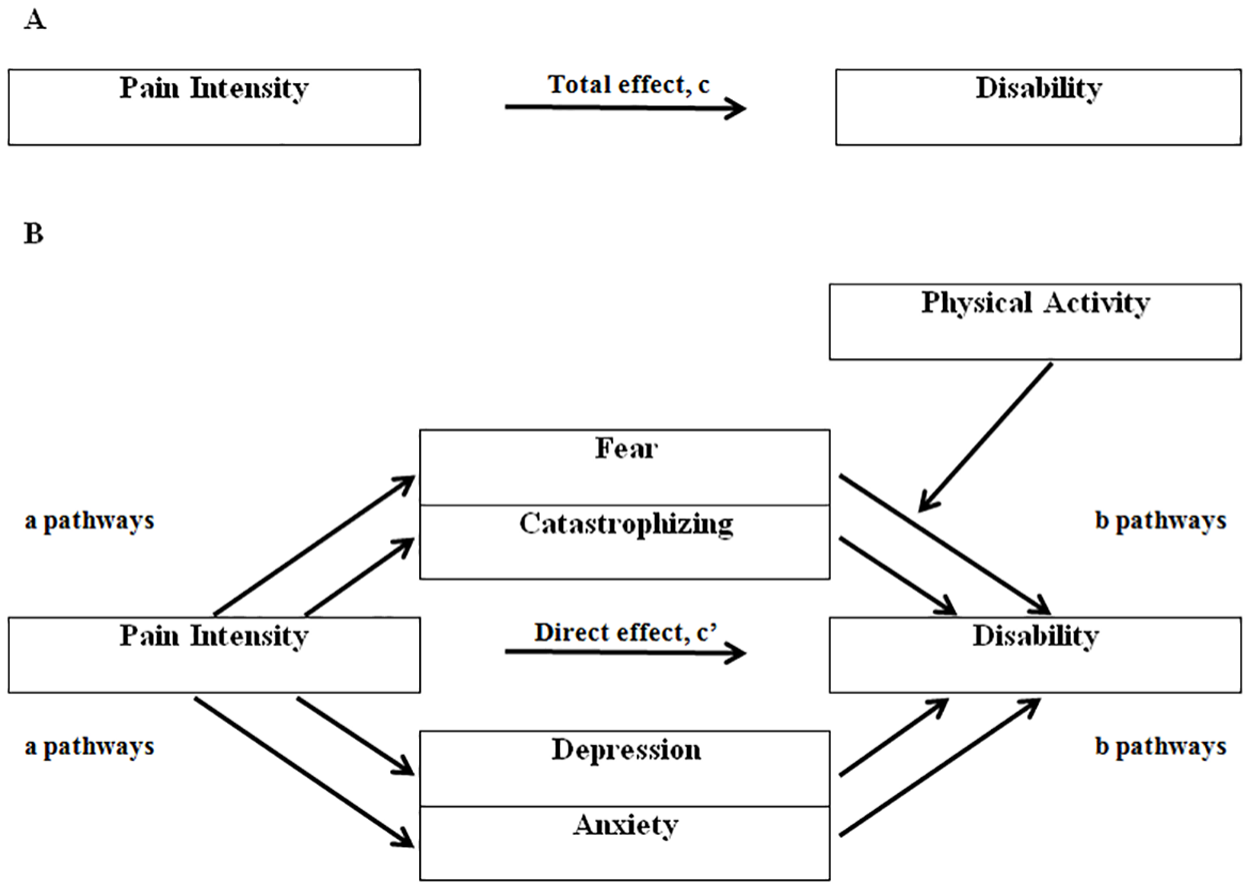
Example of the mediation-moderation model tested in this study. (A) is the primary relationship between pain and disability, with the total effect labelled c. (B) are the proposed mechanisms of mediation and moderation. The direct effect (c’) is the effect of pain on disability after controlling for the mediator variables. The indirect effect of pain on the mediators are the ‘a pathways’. The indirect effect of the mediators on disability are the ‘b pathways’.

**Fig 2:**
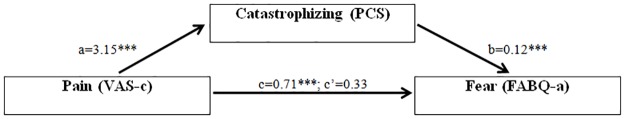
Mediation of the relationship between pain and fear through catastrophizing. Coefficients for the different pathways (a, b, c) are displayed. The indirect effect (a x b pathway) for PCS was 0.37 (95% CI 0.15 to 0.62). *** is p<0.001.

The following a priori steps had to be successfully met to confirm mediation: 1) pain was significantly associated with disability (total effect; c path, Fig 1); 2) pain was significantly associated with each of the proposed mediator variables (fear, catastrophizing, anxiety, depression; a paths), 3) controlling for pain, each of the proposed mediators was significantly associated with disability (b paths), and 4) the relationship between pain and disability was reduced (direct effect, c’ path) when controlling for the proposed mediators (indirect effect, a x b), with the 95% confidence interval (CI) for the indirect effect of each proposed mediating variable outside 0 (Fig 1).

A custom written macro (PROCESS; www.processmacro.org) was downloaded into SPSS (v22, IBM, USA) based on recommendations for how to perform multiple mediation pathway analysis with bias-corrected bootstrapping tests [34–36]. Bootstrapping is a statistical method that involves drawing repeated samples from the data with replacement in order to gain multiple estimates of the indirect effect attributed to potential mediator variables [36]. Advantages to using this statistical approach for testing mediation over Baron and Kenny’s 4-step method [39] is that it does not make the assumption of normality for the direct effects, and multiple mediators can be tested simultaneously [35,36]. Furthermore, type I error is reduced because fewer statistical tests are required [36].

Two mediation analyses were performed (PROCESS, model 4) to examine whether the proposed mediators influenced the relationship between VAS-c and VAS-w with ODI. Further analyses were performed (model 4) to examine whether PCS mediated the relationship between VAS scores and fear (FABQ-a, FABQ-w).

We tested for moderated mediation of the entire FABQ model using PROCESS model 15. First, the conditional indirect effects (a x b path) for each mediator variable were compared between individuals who did and did not report performance of a structured weekly physical activity session using bias-corrected bootstrapping (5,000 resamples). If 95% confidence intervals for the between-group contrast did not include 0 the separate indirect effects for each variable were inspected to determine which physical activity group influenced mediation outcomes. All regression coefficients are presented as the unstandardized regression coefficients (*B*) from the PROCESS macro. The significance level of this study was p ≤ 0.05.

## Results

### Relationship between variables

All correlation coefficients were below 0.90 indicating that multicollinearity was not present (Table 2). ODI and VAS-c were associated with all variables, and VAS-w was associated with all variables apart from HADS-a. Therefore the only variable excluded from mediation analyses was for HADS-a in the relationship between VAS-w and ODI.

### Mediation of the relationship between pain and disability

The mediation analyses (Tables 3 and 4) revealed similar outcomes for current pain intensity (VAS-c) and worst pain in the last week (VAS-w), therefore only the VAS-c result will be clarified further. The overall regression model showed that 46.9% (p<0.001) of the variance in ODI scores was explained by VAS-c and the mediator variables. The relationship between pain (VAS-c) and disabilty (ODI) was significant (total effect, c pathway, *B* = 3.02, r^2^ = 0.26, p<0.001). The overall indirect effect for the multiple mediator model was *B* = 1.50 (95% CI = 0.88 to 2.16), and accounted for 49.7% of the total effect. Indirect effects for the proposed mediators are depicted in Table 4. Only FABQ-a, PCS, and HADS-d met all criteria for significant mediation of the relationship between pain and disability. Overall, 42.4% of the variability in the relationship between pain and disability was explained by FABQ-a, PCS, and HADS-d.

**Table 3:** Total effect, direct effect, indirect effect, and r^2^ values for the mediation models of current pain (VAS-c) and worst pain in the last week (VAS-w) with disability (ODI).

Model	Path	*B*	95% CI	SE	t score	p-value	Model r^2^
VAS-c to ODI	Total effect (c)	3.02	2.35 to 3.70	0.34	8.82	<0.001	0.26
	Direct effect (c’)	1.51	0.79 to 2.24	0.37	4.09	<0.001	
	Indirect effect (a x b)	1.50	0.88 to 2.16	0.33			
VAS-w to ODI	Total effect (c)	2.42	1.79 to 3.06	0.32	7.55	<0.001	0.21
	Direct effect (c’)	1.07	0.45 to 1.68	0.31	3.43	<0.001	
	Indirect effect (a x b)	1.36	0.92 to 1.92	0.25			

**Table 4:** Indirect paths of the multiple mediator model for fear (FABQ-a, FABQ-w), catastrophizing (PCS), depression (HADS-d), and anxiety (HADS-a). 95% confidence intervals for the indirect effect were calculated using bias-corrected bootstrapping with 5,000 resamples.

	a Path (pain on mediator)				b path (mediator on disability)				Indirect effect (a x b path)		
	*B*	SE	t score	p-value	*B*	SE	t score	p-value	*B*	SE	95% CI
VAS-c to ODI											
FABQ-a	0.71	0.16	4.49	<0.001	0.72	0.13	5.43	<0.001	0.51	0.15	0.25 to 0.85
FABQ-w	1.03	0.28	3.68	<0.001	-0.02	0.08	-0.21	0.832	-0.01	0.08	-0.22 to 0.12
PCS	3.14	0.30	10.45	<0.001	0.22	0.08	2.80	0.006	0.70	0.30	0.11 to 1.34
HADS-a	0.20	0.10	2.03	0.043	0.21	0.25	0.85	0.399	0.04	0.06	-0.05 to 0.21
HADS-d	0.42	0.11	3.96	<0.001	0.64	0.26	2.48	0.014	0.27	0.16	0.02 to 0.65
VAS-w to ODI											
FABQ-a	0.69	0.14	4.87	<0.001	0.70	0.14	5.12	<0.001	0.48	0.13	0.25 to 0.75
FABQ-w	0.57	0.26	2.23	0.027	0.02	0.08	0.22	0.823	0.01	0.05	-0.08 to 0.11
PCS	2.32	0.29	7.91	<0.001	0.29	0.08	3.79	<0.001	0.67	0.21	0.29 to 1.13
HADS-d	0.28	0.10	2.88	0.004	0.63	0.26	2.43	0.016	0.18	0.12	0.02 to 0.49

### Catastrophizing as a mediator of the relationship between pain and fear

Because of the similarity in outcomes between VAS-c and VAS-w (Tables 3 and 4), and that only FABQ-a was a significant mediator of the relationship between pain and disability (Table 4), we only tested whether PCS mediated the significant relationship between VAS-c and FABQ-a (*B* = 0.71, r^2^ = 0.09, p<0.001). All criteria for significant mediation were met (Fig 2), with the indirect effect of PCS (*B* = 0.37, 95% CI 0.15 to 0.62) explaining 53% of the total effect of VAS-c on FABQ-a.

### Physical activity moderates the effect of catastrophizing

The indirect effect of catastrophizing on the relationship between VAS-c and ODI was significantly moderated by reporting of weekly structured physical activity (Table 5). Specifically, the indirect effect for PCS mediating the relationship between VAS-c and ODI was only significant for individuals reporting weekly physical activity (*B* = 1.31, 95% CI 0.44 to 2.23), compared to individuals reporting no weekly physical activity (*B* = 0.21, 95% CI -0.50 to 0.97).

**Table 5:** Test of equality between the moderated indirect effects (a x b pathway) for patients with low back pain who did and did not report weekly physical activity, using bias-corrected bootstrapping (5,000 resamples).

	*B*	SE	95% CI
VAS-c to ODI			
FABQ-a	0.24	0.21	-0.11 to 0.73
FABQ-w	-0.09	0.16	-0.45 to 0.22
PCS	-1.10	0.57	-2.31 to -0.32
HADS-a	0.03	0.10	-0.14 to 0.28
HADS-d	0.32	0.26	-0.11 to 0.92
VAS-w to ODI			
FABQ-a	0.18	0.18	-0.12 to 0.59
FABQ-w	-0.05	0.10	-0.28 to 0.10
PCS	-0.63	0.39	-1.48 to 0.11
HADS-d	0.21	0.17	-0.03 to 0.64

The conditional indirect effects for PCS mediating the relationship between VAS-c and FABQ-a were not different between individuals who did and did not report weekly physical activity (*B* = 0.37, 95% CI -0.17 to 0.94).

## Discussion

### Summary of main findings

We conducted this study to investigate proposed pathways within the fear-avoidance model in a relatively large sample (n = 218) of chronic low back pain patients, and to address gaps in the literature pertaining to the potential mediating effect of catastrophizing on the pain-disability relationship. Moreover this was the first study to examine the moderating effect of physical activity on pathways within the fear-avoidance model. The main findings of this study were 1) fear, catastrophizing, and depression explained 42.4% of the relationship between pain and disability in patients with chronic low back pain, 2) the mediating effect of catastrophizing was conditional upon the performance of weekly structured physical activity, and 3) catastrophizing mediated the relationship between pain and fear, the first proposed pathway in the fear-avoidance model, and this was not conditional upon the performance of regular physical activity.

### Fear-avoidance

The results of this study support previous findings for the role of fear-avoidance and depression as significant mediators of the positive relationship between pain and disability in chronic back pain patients [11]. Thus the relationship between higher pain and disability is, in part, explained by higher self-rated fear-avoidance beliefs about physical activity and depression. A novel finding of this study was that the mediating effect of fear was not conditional upon physical activity. This provides insight into the confusing findings with regards to changes in fear-avoidance following physical activity interventions. While some studies have reported small-to-medium effect sizes for reductions in fear following physical activity or exercise interventions for back pain [40–43], a number of studies have shown no change in measures of fear-avoidance despite reduced pain and disability [44–48]. These equivocal findings lead to confusion for evidence-based practitioners attempting to understand why a physical activity based intervention may or may not be effective for reducing fear-avoidance beliefs in chronic back pain patients. Recent evidence suggests that physical activity interventions are only effective for reducing high fear-avoidance beliefs when combined with cognitive behavioural approaches [49,50]. The results of this study provide support for the need to supplement physical activity interventions with cognitive approaches, because the mediating effect of fear on pain related disability was not conditional upon performing weekly structured physical activity. In other words, performing exercise alone is likely not sufficient to reduce fear of movement and therefore pain related disability in people with chronic low back pain.

### Catastrophizing

We have also extended current understanding for the role of catastrophizing as a mediator of the relationship between pain and disability in chronic back pain patients. Catastrophizing is defined as an exaggerated negative interpretation of pain that may occur during an actual or anticipated pain experience [27]. There are equivocal results for the association between catastrophizing and pain related disability in back pain patients [9,51–54], and for catastrophizing as a factor to explain successful outcomes in back pain patients following different types of treatment [13,47,55,56]. The pooled coefficient from the recent meta-analysis [11] for the indirect effect (a x b pathway) of catastrophizing as a mediator of the pain-disability relationship was not significant (*B* = 0.07, 95% CI -0.06 to 0.19), although based on a relatively small sample (3 studies, n = 234 patients) and inclusive of both acute and chronic back pain patients. Our analyses of 218 people with chronic back pain revealed that the indirect effect of catastrophizing, but not fear or depression, was conditional upon reporting engagement with weekly structured physical activity (conditional a x b pathway; *B* = 1.31, 95% CI 0.44 to 2.23). Thus in people with chronic back pain who reported weekly physical activity, albeit within our definition of physical activity, higher catastrophizing scores in addition to fear and depression explained the relationship between pain and disability. Catastrophizing had no influence on the pain-disability relationship for chronic back pain patients who reported no weekly structured physical activity, with an indirect effect comparable to previous data (*B* = 0.21, 95% CI -0.50 to 0.97). Therefore a unique and important recommendation for clinical practice is that people with chronic back pain who regularly engage with, or potentially initiate regular physical activity, may require specific psychological counselling or support with regards to negative perceptions about pain. Because this was a cross-sectional study, the temporal relationship between performing regular physical activity and negative pain perceptions are unclear.

The second objective of this study was to examine the proposed pathway of the fear-avoidance model where catastrophizing mediates the relationship between pain and fear. To our knowledge, we are the first to report that catastrophizing is a significant, positive mediator of the relationship between pain and fear in chronic low back pain patients, and that this relationship is not conditional on physical activity. While significant, the relationship between pain and fear was relatively small (r^2^ = 0.09). Therefore the relative importance of this pathway should be questioned. Indeed two prospective studies [57,58] showed that early changes in catastrophizing after injury or following early engagement with a treatment provider for musculoskeletal pain do not precede changes in fear, or predict changes in disability or depression. Therefore while a statistically significant finding, the clinical relevance of catastrophizing as a mediator of the relationship between fear and pain seems limited.

### Implications for practice

Recently the fear-avoidance model has been critiqued for both the lack of empirical support for proposed pathways, or consideration for how multi-dimensional processes (e.g. social, cultural, environmental factors) influence relationships [59]. For clinicians, the relative importance of the fear-avoidance model is often discussed, but translating research into effective treatment for people with chronic low back pain is lacking. Our findings are novel because they show that an external condition, in this case performance of weekly structured physical activity, explains relationships between proposed belief pathways in the fear-avoidance model. Indeed a strength of our data analysis is that mediation is often thought to reveal specific variables to be targeted with interventions, such as fear and depression. Our data supports current recommendations that psychological counselling with regards to fear and depression should be a standard treatment inclusion for people with chronic low back pain [11,49].

The results of our study also provide unique clinical perspectives with regards to the relationship between regular physical activity, catastrophizing, and fear-avoidance. First, people with chronic low back pain who engage with weekly physical activity appear to require additional support to address negative pain perceptions. What this support entails is unclear from the current study, although education about chronic pain (e.g. [49]) as compared to the feelings of discomfort elicited from normal physical activity is a likely first step. Second, it appears that engagement with regular physical activity is not necessary to influence the mediating effect of fear-avoidance beliefs on the pain-disability relationship. While physical activity interventions have tremendous benefits for overall health and are frequently prescribed in chronic back pain, the overall effect size for these interventions on back pain related disability is small-to-medium [60,61]. Our data suggest that greater emphasis may need to be placed on the psychosocial components of pain to complement and improve the response to physical activity interventions.

### Limitations

There are several limitations that should be considered. The definition of weekly physical activity was based on the self-report of at least one session per week for the last month consisting of either cardiorespiratory type exercise, trunk exercise (self- or therapist-directed), or other forms of physical activity. This definition does not quantify physical activity in terms of gross caloric expenditure, nor provide activity ‘dose’ information. Quantification of physical activity based on accelerometer data would not accurately capture exercise routinely performed by back pain patients, such as a trunk stability program that involves minimal whole body movement (i.e. accelerations). We did not elect to categorize physical activity based on a higher threshold, such as three times per week, based on the current low back pain rehabilitation literature where one session per week appears equitable to a minimum level likely to have positive outcomes for patients [60]. Nor did we want to compare different types of exercise, since the overwhelming evidence is that no one mode of exercise is superior to any other for chronic back pain rehabilitation [60].

With regards to the mediation analyses conducted in this study, the explanatory factors of fear, depression, and catastrophizing did not completely mediate the relationship between pain and disability. There are likely other behavioural factors, such as self-efficacy, that contribute to this relationship [11]. However, the scope of this study was with regards to pathways described within the fear-avoidance model. While ongoing discussion in the literature attempts to refine and update this model, we did not measure variables that as yet are not typically included within the fear-avoidance pathways.

The results of this study should not be generalized to all back pain patients. In particular fear about work related activities, as well as depression and anxiety scores, were lower than reported in other studies of chronic back pain patients. These lower scores may, in part, explain why FABQ-w and HADS-a scores were not identified as significant mediators. However, we believe our sample is representative of the typical patient who chooses to engage with treatment. Indeed scores for disability, pain, and fear about physical activity were similar to baseline values for recent clinical trials [47,49]. Thus our findings likely have good generalizability for clinical practice.

Based on estimates for required sample sizes in mediation analyses [17], our study was not sufficiently powered to detect significant indirect effects when the a and b paths (exposure to mediator, mediator to outcome respectively) were small (*B* = 0.14). For example a small ‘a path’ but large ‘b path’ (*B* = 0.60) is suggested to require n = 365. Inspection of our data (Table 4) would suggest that we were only underpowered to detect a significant indirect effect for anxiety (HADS-a) mediating the relationship between current pain and disability. However, further exploration of these pathways is needed.

Finally, while we designed this study to address many of the quality recommendations for mediation analyses (e.g. a theoretical framework, sample size justification, accurate pathway analysis and inspection of indirect effects, [11]), we were unable to address temporal causality for relationships between the respective variables (i.e. physical activity and catastrophizing). This study was a cross-sectional examination of participants with chronic low back pain from the local community who attended the University research facility for different experimental studies. Therefore no inferences can be made about whether changes in one variable precede another.

## Conclusion

This study found that fear, depression, and catastrophizing mediate the relationship between pain and disability in people with chronic low back pain. The mediating effect of catastrophizing, but not fear or depression, was conditional upon participants reporting weekly performance of structured physical activity sessions. Thus chronic back pain patients who engage with regular physical activity may require psychological intervention and support for negative perceptions of pain. The effect of fear and depression on pain related disability was not related to regular physical activity, suggesting that psychological interventions are likely the best treatment choice for these factors.

## Supporting information

S1 Table Self report data from participants in this study. (PDF)

## Abbreviations

FABQ-a: fear-avoidance beliefs questionnaire – activity score
FABQ-w: fear-avoidance beliefs questionnaire – work score
HADS-a: Hospital Depression and Anxiety Scale – anxiety
HADS-d: Hospital Depression and Anxiety Scale – depression
LBP: low back pain
METS: metabolic equivalents
ODI: Oswestry disability index
PCS: pain catastrophizing scale
VAS-c: visual analog pain scale - current pain
VAS-w: visual analog pain scale - worse pain last week
