# Supplementary material for: Physical activity and the mediating effect of fear, depression, anxiety, and catastrophizing on pain related disability in people with chronic low back pain
Source: PLoS One. 2017 Jul 7;12(7):e0180788. doi: 10.1371/journal.pone.0180788 (PMC5501599; doi:10.1371/journal.pone.0180788)
Supplement: S1 Table — (PDF) [file pone.0180788.s001.pdf]

ODI %	HAD ANX	HADS DEP	FAB WORK	FAB PA	PCS TOTAL	VAS CURRENT
50	12	11	23	14	46	6.4
20	4	1	23	22	10	7
52	8	6	4	10	55	7.8
22	5	5	33	12	3	5
28	0	9	26	16	10	6.9
28	8	5	15	28	0	1.9
26	7	3	35	18	29	7.4
46	3	3	27	20	15	7.8
27	5	3	14	12	11	5.2
10	7	2	23	15	8	1.5
16	4	1	28	18	13	2.8
26	11	2	29	13	20	3.1
14	5	2	0	8	11	2.3
10	0	0	5	12	17	0
6	2	1	17	4	0	0.7
14	0	0	9	12	1	0.4
8	6	4	0	1	0	2.5
16	10	1	7	14	11	2.7
16	3	0	6	17	9	5.2
22	1	1	12	11	14	4
6	3	3	0	9	4	1.9
8	5	0	5	0	4	3.9
18	6	1	12	11	4	4.5
16	7	7	10	14	21	0
8	2	0	0	11	2	0.8
12	4	1	6	8	3	0
14	4	1	5	15	0	1.6
6	8	3	19	0	2	2.5
14	1	3	8	11	3	0.5
10	13	2	7	10	2	0
8	5	1	4	8	2	1.1
10	1	2	0	14	1	1.1
18	7	8	0	4	29	1.9
16	6	1	0	6	6	3.5
54	5	3	4	18	6	1.9
10	7	1	0	17	8	1.8
12	0	2	9	9	5	1
12	3	2	9	12	6	0.9
10	3	2	13	13	9	1.7
10	7	7	24	4	0	2.5
26	3	1	10	20	8	2.3
20	8	4	12	13	12	2.6
12	12	10	0	14	3	6.2
22	10	1	0	15	4	4.3
6	0	2	0	14	3	1.3
22	2	3	22	24	14	3.8
14	8	8	7	11	6	2
14	7	1	7	0	4	0
14	2	2	1	16	8	2.3

12	3	4	0	19	9	5.5
8	8	0	0	3	2	0.3
16	8	5	12	13	15	0.4
18	5	1	18	12	5	1
12	6	2	19	6	15	2.8
6	6	6	3	7	4	2.5
6	8	8	0	0	0	0.2
30	6	6	2	8	1	1.5
12	4	4	0	12	8	1
10	2	1	1	8	13	2.3
16	1	3	12	13	1	2.5
16	14	8	23	14	25	1.4
34	2	0	11	14	22	3.1
20	4	2	10	13	14	1.3
26	10	9	10	15	35	3.6
14	8	8	7	18	1	0
12	3	2	16	12	4	1.4
26	11	2	29	13	20	3.1
22	7	7	20	6	14	5.2
20	9	15	30	16	15	0.4
22	5	2	35	18	5	1.6
14	0	0	21	14	0	3.8
26	3	1	28	10	8	2.7
26	7	1	30	7	14	6.6
50	12	12	19	19	37	4.3
42	12	9	23	17	21	3.3
48	8	11	22	17	21	3.2
34	2	2	0	24	43	6
38	14	12	36	16	41	6.9
18	10	7	0	12	33	6.6
26	8	1	17	17	9	6.6
52	7	2	0	24	43	5.1
36	7	4	22	19	29	7.5
58	9	4	2	17	45	6.8
30	7	2	4	13	3	4.9
16	2	0	6	11	10	6.1
36	5	2	2	21	15	7.9
50	5	3	14	22	29	6.5
42	4	5	16	16	31	7.4
44	9	6	11	5	5	6.6
28	2	2	0	24	48	8.4
40	7	8	9	9	31	6.9
36	5	9	6	12	56	7.1
30	9	5	18	19	16	2.4
32	6	3	12	22	16	3.8
34	6	5	42	10	17	3.5
36	9	11	23	15	27	4.1
24	2	1	21	21	13	4.6
28	7	2	24	15	17	3.2
44	11	9	18	16	37	5.6

54	15	18	38	21	42	4.4
38	14	16	35	19	41	3.9
62	10	18	31	21	40	5.7
42	9	10	31	20	28	5.9
24	6	4	27	14	18	8.5
38	4	3	24	21	35	7.9
46	6	5	14	16	45	7.9
20	7	5	24	6	17	4.6
14	1	2	0	13	8	0
30	7	13	22	21	38	8.7
30	13	9	8	24	38	6.4
56	4	11	20	19	34	7.1
32	6	11	14	17	47	7.9
32	6	2	3	19	18	8.4
32	6	2	3	19	18	8.4
70	4	17	23	21	37	6.9
26	11	7	18	12	34	2.9
26	11	7	18	12	34	2.9
14	5	0	20	15	25	5.8
34	11	8	10	20	31	6.3
34	12	14	21	13	35	5.1
12	12	4	2	0	10	4.2
24	10	4	18	15	24	7.1
42	7	3	5	11	1	0.9
12	2	1	3	10	16	0.7
24	9	2	9	11	8	1.6
10	3	0	9	15	3	1.3
36	5	2	3	15	2	1.4
64	6	3	0	14	3	1.2
26	12	9	5	9	9	2.8
20	8	9	1	16	16	1.2
20	7	2	5	19	19	3.7
18	8	6	0	13	16	2.5
30	2	1	13	19	20	3.2
20	2	1	4	15	4	1.5
20	8	8	2	5	30	2.5
14	9	10	6	13	27	2
52	9	3	3	18	18	3.4
18	5	3	9	17	28	1.3
14	6	4	0	18	17	1
48	8	6	0	16	32	4.2
18	6	1	5	13	27	1.5
40	5	2	6	18	5	2
16	5	0	10	16	4	1.9
14	4	0	5	11	5	5
30	2	3	1	4	0	3.9
34	3	1	6	24	15	5.2
28	1	1	17	17	5	4.8
16	1	1	19	9	2	4.1
16	5	1	0	13	12	2.7

20	7	6	17	13	11	5.7
22	12	3	0	13	6	1.6
40	0	3	5	13	0	2
36	1	3	3	19	22	4
28	3	1	19	15	10	1.8
22	7	3	5	16	7	2.7
38	4	2	7	23	12	3.8
26	5	3	7	18	9	4.5
14	7	2	0	4	10	1.6
38	8	2	3	15	5	1.6
50	9	6	6	11	19	5.4
16	5	5	0	15	21	3.1
32	10	5	20	17	22	7
28	3	2	13	13	22	4.2
28	7	4	8	24	18	3.2
32	7	5	7	7	10	6.7
62	10	2	0	24	35	5.8
38	3	2	0	22	12	7.4
12	7	5	0	4	10	3.7
46	5	2	0	23	28	6.2
26	2	5	12	17	19	6.7
12	9	3	12	7	9	2.5
42	4	2	8	21	8	2.5
42	4	2	8	21	8	2.5
14	2	2	17	10	13	6.3
12	1	3	15	19	8	2.7
34	7	2	3	17	12	5
22	4	10	9	14	12	1.3
16	10	1	12	21	6	2.4
10	6	2	5	7	2	0.3
8	3	1	1	8	13	0
10	2	2	10	9	1	1
16	1	1	14	12	5	0.9
12	4	4	2	4	9	2.4
18	7	4	8	12	8	2.3
38	9	6	8	16	16	3.6
8	8	4	5	10	5	1.4
14	14	11	19	14	14	3.5
22	3	1	18	16	12	1.7
10	0	0	0	18	0	3.1
18	9	3	27	10	0	1.4
22	4	5	13	14	5	3.8
12	6	3	1	20	5	0.9
20	0	5	16	11	17	1.9
12	5	1	16	17	5	1.3
14	0	0	9	5	4	3
58	7	3	0	19	23	3.2
20	5	1	3	16	14	3.4
10	3	0	5	20	19	4.1
32	8	5	18	14	28	6.3

26	7	4	14	14	14	0.4
24	1	3	8	10	25	5.9
14	7	3	10	5	14	4
46	15	15	42	18	37	5
35	7	7	9	1	17	8.4
10	6	0	12	12	0	2
32	10	12	7	7	28	6.3
20	5	6	12	14	15	4.1
18	6	3	7	18	14	2.7
14	4	1	0	18	15	1.9
18	11	7	16	8	25	1.8
30	4	2	26	8	25	6.2
16	2	2	10	8	2	1
12	7	3	3	14	20	3.1
38	6	6	13	9	24	7.9
18	8	7	0	20	25	6.6
26	4	1	25	19	29	6.8
32	9	6	6	20	20	3.9
16	6	6	15	9	20	2.5

VAS WORST	. woman, 2	PA 1 yes,	MEDS 1 yes, 2 no
7.5	1	1	1
8.7	1	1	1
9	1	1	1
5.7	1	1	1
4.7	1	1	1
7.8	1	1	1
8.6	1	1	1
9.4	1	1	1
8	1	1	1
1.5	2	1	1
2.4	2	1	1
3.4	2	1	1
8.5	2	1	1
0	1	1	2
0.9	1	1	2
1	1	1	2
4.1	1	1	2
4.2	1	1	2
5.2	1	1	2
5.8	1	1	2
5.8	1	1	2
6.1	1	1	2
7.2	1	1	2
0	1	1	2
1.8	1	1	2
0.8	1	1	2
1.8	1	1	2
4.3	1	1	2
0.4	1	1	2
0.8	1	1	2
3	1	1	2
3.4	1	1	2
3.9	1	1	2
6.6	1	1	2
5.3	1	1	2
1.6	1	1	2
2.7	1	1	2
3.3	1	1	2
4.7	1	1	2
6	1	1	2
6.3	1	1	2
6.8	1	1	2
7	1	1	2
7.2	1	1	2
1.3	1	1	2
5	1	1	2
3	1	1	2
3.5	1	1	2
4.4	1	1	2

5.5	1	1	2
0.4	2	1	2
2.5	2	1	2
6.7	2	1	2
6.8	2	1	2
6.9	2	1	2
0.2	2	1	2
3.1	2	1	2
2.5	2	1	2
2.3	2	1	2
2.5	2	1	2
3.1	2	1	2
3.2	2	1	2
3.6	2	1	2
5.5	2	1	2
1.4	2	1	2
2.4	2	1	2
3.4	2	1	2
7.2	2	1	2
2.5	1	2	1
2.6	1	2	1
3.6	1	2	1
3.7	1	2	1
5.5	1	2	1
5.8	1	2	1
6.5	1	2	1
6.6	1	2	1
6.6	1	2	1
7	1	2	1
7	1	2	1
7.9	1	2	1
8	1	2	1
8.1	1	2	1
8.3	1	2	1
8.5	1	2	1
8.5	1	2	1
8.6	1	2	1
8.7	1	2	1
8.9	1	2	1
9.1	1	2	1
9.2	1	2	1
9.5	1	2	1
9.9	1	2	1
10	1	2	1
7.9	1	2	1
4	2	2	1
4.1	2	2	1
4.4	2	2	1
4.9	2	2	1
5.9	2	2	1

6.7	2	2	1
7	2	2	1
8.2	2	2	1
8.2	2	2	1
8.4	2	2	1
8.9	2	2	1
9	2	2	1
9	2	2	1
9	2	2	1
9.1	2	2	1
9.2	2	2	1
9.3	2	2	1
9.5	2	2	1
9.6	2	2	1
9.6	2	2	1
9.7	2	2	1
9.8	2	2	1
9.8	2	2	1
10	2	2	1
4.1	2	2	1
6.7	2	2	1
0	1	2	2
0.3	1	2	2
1.6	1	2	2
1.7	1	2	2
1.9	1	2	2
2.1	1	2	2
2.2	1	2	2
2.6	1	2	2
2.7	1	2	2
2.7	1	2	2
3.5	1	2	2
3.7	1	2	2
3.9	1	2	2
4.2	1	2	2
4.5	1	2	2
4.5	1	2	2
4.6	1	2	2
4.8	1	2	2
4.8	1	2	2
4.9	1	2	2
4.9	1	2	2
5.1	1	2	2
5.3	1	2	2
5.4	1	2	2
5.8	1	2	2
5.9	1	2	2
5.9	1	2	2
5.9	1	2	2
6	1	2	2

6.2	1	2	2
6.3	1	2	2
6.4	1	2	2
6.5	1	2	2
6.6	1	2	2
6.8	1	2	2
6.9	1	2	2
6.9	1	2	2
6.9	1	2	2
6.9	1	2	2
7	1	2	2
7.1	1	2	2
7.2	1	2	2
7.2	1	2	2
7.2	1	2	2
7.3	1	2	2
7.6	1	2	2
7.6	1	2	2
7.6	1	2	2
7.8	1	2	2
7.8	1	2	2
7.9	1	2	2
8	1	2	2
8	1	2	2
8	1	2	2
8.1	1	2	2
8.3	1	2	2
1.2	1	2	2
5.9	1	2	2
0.8	2	2	2
1	2	2	2
1	2	2	2
1.4	2	2	2
2.4	2	2	2
2.7	2	2	2
2.9	2	2	2
3.2	2	2	2
3.4	2	2	2
3.4	2	2	2
3.4	2	2	2
3.7	2	2	2
3.8	2	2	2
3.8	2	2	2
4.6	2	2	2
4.8	2	2	2
4.9	2	2	2
5.3	2	2	2
5.3	2	2	2
5.3	2	2	2
5.6	2	2	2

5.6	2	2	2
5.9	2	2	2
5.9	2	2	2
6	2	2	2
6	2	2	2
6	2	2	2
6.4	2	2	2
6.5	2	2	2
6.7	2	2	2
6.9	2	2	2
7	2	2	2
7.1	2	2	2
7.2	2	2	2
7.4	2	2	2
7.5	2	2	2
7.9	2	2	2
8	2	2	2
5.7	2	2	2
6	2	2	2
